# Cytokine-Supplemented Maturation Medium Enhances Cytoplasmic and Nuclear Maturation in Bovine Oocytes

**DOI:** 10.3390/ani14121837

**Published:** 2024-06-20

**Authors:** Renata Blocher, Ying Liu, Tayler Patrick, Irina A. Polejaeva

**Affiliations:** Animal, Dairy, and Veterinary Sciences Department, Utah State University, 4815 Old Main Hill, Logan, UT 84322, USA; renata.hoskova@usu.edu (R.B.); ying.liu@usu.edu (Y.L.); tayler.patrick@usu.edu (T.P.)

**Keywords:** in vitro maturation, cytokines, FLI, bovine oocytes, meiotic spindle, mitochondria, cortical granules

## Abstract

**Simple Summary:**

Oocytes, the female reproductive cell, must undergo various DNA (nuclear) and cytoplasmic changes called maturation. Oocyte maturation in vitro is not as efficient as within the animal. Supplementation of three cytokines (FLI) previously increased oocyte maturation and subsequent embryo development. However, studies have not explored how FLI supplementation affects specific important aspects of maturation. This study assessed nuclear and cytoplasmic events associated with high-quality oocytes in FLI and control (BMM) oocytes. FLI-supplemented oocytes progressed through meiosis I (DNA changes) more than BMM, as indicated by an increase in nuclear maturation rate, normal meiotic spindles, and telophase I incidence. Additionally, FLI-treated oocytes had better mitochondrial and cortical granule (CG) distribution after maturation, with more oocytes exhibiting a diffuse mitochondrial and pattern I CG distribution. The data suggest that FLI-supplementation of oocytes during maturation coordinates nuclear and cytoplasmic maturation to produce higher-quality oocytes. It may be used in the future to increase the efficiency of assisted reproductive technologies for livestock and humans.

**Abstract:**

Bovine in vitro oocyte maturation (IVM) is an easy way to obtain oocytes for subsequent assisted reproductive techniques but is inefficient compared to in vivo maturation. Supplementation of three cytokines, fibroblast growth factor 2 (FGF2), leukemia inhibitory factor (LIF), and insulin-like growth factor 1 (IGF1), or FLI, has increased oocyte maturation and embryo development in multiple species, but studies have not explored the oocyte differences caused by FLI IVM supplementation. This study aimed to assess important nuclear and cytoplasmic maturation events in high-quality oocytes. FLI-supplemented oocytes had a decreased GV (3.0% vs. 13.7%, *p* < 0.01) and increased telophase I incidence (34.6% vs. 17.6%, *p* < 0.05) after IVM, increased normal meiotic spindles (68.8% vs. 50.0%, *p* < 0.001), and an increased nuclear maturation rate (75.1% vs. 66.8%, *p* < 0.001). Moreover, in metaphase II oocytes, the percentage of FLI-treated oocytes with a diffuse mitochondrial distribution was higher (87.7% vs. 77.5%, *p* < 0.05) and with a cortical mitochondrial distribution was lower (11.6% vs. 17.4%, *p* < 0.05). Additionally, FLI-supplemented oocytes had more pattern I cortical granules (21.3% vs. 14.4%, *p* < 0.05). These data suggest that FLI supplementation in bovine in vitro maturation medium coordinates nuclear and cytoplasmic maturation to produce higher-quality oocytes.

## 1. Introduction

When compared to in vivo maturation, in vitro maturation (IVM) of livestock oocytes is an inexpensive and easy way to obtain mature oocytes for assisted reproductive technologies (ARTs), such as in vitro fertilization (IVF) and somatic cell nuclear transfer (SCNT). However, IVM is not as efficient as in vivo maturation and produces oocytes with lower developmental competency due to changes in gene expression and increased environmental stress [1,2]. Research focus has been studying the supplementation of various compounds in IVM media to increase maturation rates and developmental potential [3,4,5].

Cytokines play a critical role in in vivo folliculogenesis by regulating cellular proliferation/differentiation, follicular survival/atresia, and oocyte maturation [6]. Among others, the following three cytokines found in follicular fluid have been added to IVM media of various species: fibroblast growth factor 2 (FGF2), leukemia inhibitory factor (LIF), and insulin-like growth factor 1 (IGF1). Each cytokine has beneficial effects on in vitro maturation that are amplified when the cytokines are added in combination. FGF2 increases cumulus cell expansion [7], LIF increases oocyte maturation and early embryonic development [8], and IGF1 increases oocyte quality and embryonic development [9,10]. Researchers investigated the effects of maturation medium supplementation with these three cytokines, termed FLI, in the porcine model. FLI supplementation at 40 ng/mL FGF2, 20 ng/mL LIF, and 20 ng/mL IGF1 increased porcine oocyte maturation and live offspring rates after IVF and embryo transfers [11]. In our previous report, FLI supplementation in bovine IVM at the above concentrations improved oocyte maturation, blastocyst development following IVF and SCNT, and live offspring rates following SCNT and embryo transfers [12]. The addition of FLI at the previously tested concentrations in the maturation medium also increases IVM in mice [13] and humans [14]. Thus, FLI supplementation increases in vitro oocyte maturation and embryo production in numerous species.

The two main aspects of in vitro maturation, cytoplasmic and nuclear maturation, must occur simultaneously to produce oocytes capable of developing well during in vitro culture [15,16,17]. Cytoplasmic and nuclear maturation are defined as the rearranging of organelles and chromosomes within the oocyte, respectively. Various components of cytoplasmic and nuclear maturation have been studied and linked to oocyte developmental potential. Within the oocyte’s cytoplasm, mitochondria are an important element as they provide the energy needed for both oocyte maturation and embryonic development up to the maternal-to-embryonic transition, or the 8-cell stage in cattle [18]. Mitochondria must reorganize from cortical in the germinal vesicle stage to diffuse in the metaphase II stage of the bovine oocyte [19]. Mitochondrial redistribution during maturation has been associated with better embryo development after in vitro fertilization [20]. While redistribution is occurring, mitochondrial activity increases in more competent bovine oocytes both before the resumption of meiosis I and before the completion of maturation [21]. This increase in activity may aid other cytoplasmic or nuclear maturation events. Besides mitochondria, cortical granules (CGs) are another component in the oocyte’s cytoplasm that must redistribute during oocyte maturation. The main role of CGs is to block polyspermy during in vitro fertilization. CGs follow an inverse pattern to mitochondria, starting as diffuse and organizing in large clusters in the oocyte cortex [22].

In addition to the above specific cytoplasmic maturation events, the oocyte must resume and complete meiosis I (MI) and arrest at metaphase of meiosis II (MII), forming a meiotic spindle with aligned chromosomes and barrel-shaped microtubules. This nuclear reorganization leads to the extrusion of the first polar body, which contains half of the oocytes’ genetic information, and prepares the oocyte for fertilization or ARTs, such as SCNT. Abnormalities in meiotic spindle organization could be one of the reasons behind improper chromosome segregation and embryo development. Mis-segregation of homologous chromosomes during MI, or sister chromatids during MII, is responsible for aneuploidy and pregnancy loss in humans [23]. Oocytes must coordinate these cytoplasmic and nuclear changes to become developmentally competent, as both cytoplasmic and nuclear maturation are imperative to subsequent embryonic development [24].

Even though our previous study reported that FLI supplementation in IVM medium has improved in vitro embryo production by increasing IVM, IVF, and SCNT efficiency, research has not explored the changes FLI induces in bovine oocytes during IVM to improve their competency. This study aimed to investigate how FLI supplementation affects specific nuclear and cytoplasmic maturation events known to be important for attaining developmental competency, with the hypothesis being that oocytes matured in FLI-supplemented media coordinate nuclear and cytoplasmic maturation to increase their developmental potential both in vivo and in vitro.

## 2. Materials and Methods

All reagents were purchased from Sigma-Aldrich (St. Louis, MO, USA) unless otherwise specified. All fluorescent images were evaluated using the ImageJ image processing software version 1.53k (National Institute of Health, Bethesda, MD, USA).

### 2.1. Oocyte Collection and In Vitro Maturation

Bovine ovaries were obtained from a local abattoir (JBS, Hyrum, UT, USA) and transported to the laboratory at 25 °C in a 0.9% saline solution. Cumulus-oocyte complexes (COCs) were collected by aspiration of three to eight mm follicles using an 18-gauge needle vacuum system [12]. Grade 1 oocytes with a uniform cytoplasm and compact cumulus cells were used for in vitro maturation in a 4-well plate containing 500 µL of maturation medium and 40 COCs in each well. COCs were cultured at 38.5 °C with 5% CO_2_ in maximum humidity for 21 h in either standard bovine maturation medium (BMM) (M199 [Gibco, Billings, MT, USA], containing 10% FBS [HyClone, Logan, UT, USA], 0.5 µg/mL FSH and LH [ProSpec, Rehovot, Israel], and 100 IU/mL penicillin/streptomycin [Gibco, Billings, MT, USA]) or maturation medium supplemented with 40 ng/mL FGF2 [Peprotech, Cranbury, NJ, USA], 20 ng/mL LIF [Stemcell, Vancouver, BC, Canada], and 20 ng/mL IGF1 [Peprotech, Cranbury, NJ, USA] (FLI). Polar body extrusion, which is indicative of metaphase II, was assessed after the removal of cumulus cells with 0.3 mg/mL hyaluronidase. Oocytes were divided into four groups based on polar body presence: FLI MII (FM), FLI not MII (FNM), control MII (CM), and control not MII (CNM). All four groups were used for subsequent staining, unless otherwise noted.

### 2.2. Nuclear Maturation

#### Immature Oocyte Nuclear Staining

Immature oocytes, which are oocytes with no polar body at 21 h post-maturation, in the FNM and CNM groups were stained to assess the stage of meiosis I at which nuclear maturation arrest took place during IVM in either FLI-supplemented or standard bovine maturation media. Oocytes were denuded well to ensure no cumulus cells were present on the zona pellucida, fixed in 4% paraformaldehyde (PFA) (ThermoFisher, Waltham, MA, USA) for 20 min, and then permeabilized in PBS with 0.1% (*v*/*v*) Triton X-100 for 30 min at room temperature. After permeabilization, DNA was stained using 20 µg/mL Hoechst 33342 for 15 min in the dark. Oocytes were mounted on slides under coverslips using 3 µL Permafluor Mountant (Epridia, Kalamazoo, MI, USA). Hoechst 33342 labeling of nuclei was imaged under a fluorescent microscope (Zeiss, Baden-Württemberg, Germany) using the DAPI (blue) filter. Each oocyte was classified into one of five categories based on chromosome distribution: germinal vesicle (GV), germinal vesicle breakdown (GVBD), metaphase I, anaphase I, or telophase I [25].

### 2.3. Meiotic Spindle Staining

FM and CM oocytes were incubated for 30 min at 38.5 °C in 10 µM Paclitaxel (ThermoFisher, Waltham, MA, USA) in maturation media to stabilize microtubules. Zona pellucida (ZP) was removed from each oocyte using 3.3 mg/mL pronase in PBS (with Ca and Mg) containing 0.1% (*w*/*v*) glucose, 0.0035% (*w*/*v*) sodium pyruvate, 1% (*v*/*v*) penicillin-streptomycin, and 0.3% (*w*/*v*) BSA, fraction V. The oocytes were then fixed for 20 min using 4% PFA. ZP-free oocytes were permeabilized with PBS containing 1% (*v*/*v*) Triton X-100 for 1 h, blocked in PBS with 2% (*w*/*v*) BSA for 1 h, and incubated at 4 °C overnight in 1:200 anti-α tubulin primary antibody (A11126, Molecular Probes, Eugene, OR, USA). Oocytes were washed 3 × 15 min in PBS containing 0.05% (*v*/*v*) Tween-20 (Tw-20). A secondary antibody was applied in the dark at room temperature for 1 h using 1:500 goat anti-mouse AlexaFluor488 (A11029, Molecular Probes, Eugene, OR, USA). Both antibodies were diluted in a blocking solution. After washing for 3 × 15 min again, DNA was stained with 20 µg/mL Hoechst 33342 for 15 min in the dark. Individual oocytes were mounted in 3 µL Permaflour Mountant between two coverslips separated by double-sided tape and imaged on a Zeiss fluorescent microscope using the DAPI (blue) and GFP (green) filters. All images were taken at the same intensity setting. Each oocyte was classified into one of two groups: normal or abnormal meiotic spindle. Normal meiotic spindles consist of barrel-shaped microtubules with aligned chromosomes, and abnormal meiotic spindles consist of misshapen or misaligned microtubules with disorganized chromosomes [26]. The incidence of a normal meiotic spindle as a percentage was calculated by the number of oocytes containing a normal meiotic spindle/total number of oocytes analyzed in each group × 100.

### 2.4. Cytoplasmic Maturation

#### 2.4.1. Mitochondrial Distribution and Mass

Oocytes were incubated at 38.5 °C in 400 nM MitoTracker^®^ Green FM (Cell Signaling Technology, Danvers, MA, USA) in PBS for 30 min. After washing in PBS with 0.1% (*w*/*v*) polyvinyl alcohol (PVA), oocytes were mounted on slides and imaged with a Zeiss fluorescent microscope using the GFP (green) filter. All images were taken at the same intensity setting. Stained mitochondria within each image were grouped into one of three distributions found throughout bovine IVM: diffuse, aggregates, or cortical [27]. Mitochondrial mass for each oocyte was calculated by subtracting the average of three background intensity readings from the fluorescent intensity of the oocyte in each image using ImageJ [28,29].

#### 2.4.2. Mitochondrial Membrane Potential (MMP)

JC-1 (5,5′,6,6′-tetrachloro-1,1′, 3,3′-tetraethyl-benzimidazoyl-carbocyanine iodide) dye (Invitrogen, Waltham, MA, USA) was used to evaluate mitochondrial membrane potential using the methods described by Chen et al. [30]. Briefly, oocytes were incubated at 38.5 °C for 30 min in 10 µg/mL JC-1 was diluted in 500 µL of M199 with 10% (*v*/*v*) FBS. ZP was removed from each oocyte using the methods described above under meiotic spindle staining. ZP-free oocytes were mounted, and fluorescent images were taken with both the GFP (green fluorescence) and mRFP (red fluorescence) filters. JC-1 dye forms aggregates that fluoresce red in mitochondria with high MMP and monomers that fluoresce green in mitochondria with low MMP. The high potential (red)/low potential (green) fluorescence intensity ratio was calculated to obtain the overall mitochondrial membrane potential of each oocyte. All images were taken at the same intensity setting.

#### 2.4.3. Mitochondrial DNA Content

The mtDNA content of single oocytes was quantified using DNA extraction followed by real-time quantitative PCR (RT-qPCR). Denuded oocytes were individually frozen in 5 µL of PBS with 0.1% (*w*/*v*) polyvinyl pyrrolidine (PVP) using liquid nitrogen and stored at −80 °C. DNA extraction was performed using the QIAamp DNA micro kit (Qiagen, Hilden, Germany) according to the manufacturer’s protocol with some modifications, mainly the exclusion of the RNA carrier. DNA was eluted using 75 µL buffer AE, provided with the kit. The 12S region of bovine mitochondrial DNA was amplified using the following primers: 5′-GGG CTA CAT TCT CTA CAC CAA G-3′ (forward) and 5′-GTG CTT CAT GGC CTA ATT CAA C-3′ (reverse). A standard curve was generated using six serial dilutions with a correlation coefficient of 0.99. RT-qPCR was run in duplicate for each sample with a SYBR/ROX PCR master mix (ThermoFisher, Waltham, MA, USA), the QuantStudio3 real-time qPCR machine (ThermoFisher, Waltham, MA, USA), and the following program: hot start of 95 °C for 10 min, 95 °C for 30 s for the first cycle, and 60 °C for 15 s for the second cycle, repeated 40 times. To assess the specificity of the primers, a melting curve was run for each experiment. The sample mitochondrial DNA copy number was calculated from the equation generated from the standard curve involving the Ct value and copy number, as described in Wu et al. [31].

#### 2.4.4. Cortical Granule Distribution

Cortical granule (CG) staining was performed as described by Andreau-Vázquez et al., with modifications [32]. ZP of MII oocytes was removed using 3.3 mg/mL pronase in PBS (with Ca and Mg) containing 0.1% (*w*/*v*) glucose, 0.0035% (*w*/*v*) sodium pyruvate, 1% (*v*/*v*) penicillin-streptomycin, and 0.3% (*w*/*v*) BSA, fraction V. Oocytes were washed 5× using PBS with 0.05% (*w*/*v*) BSA. Oocytes were fixed for 20 min using 4% PFA and permeabilized using PBS with 0.05% (*w*/*v*) BSA and 0.3% (*v*/*v*) Triton X-100 for 5 min at room temperature. After washing, cortical granules were stained in the dark for 30 min using 100 µg/mL FITC-conjugated Lens Culinaris Agglutinin (LCA) (Invitrogen, Waltham, MA, USA) in PBS with 0.05% (*w*/*v*) BSA. DNA was counter-stained using 20 µg/mL Hoechst 33342 in the dark for 15 min, then oocytes were mounted and imaged with a fluorescent microscope using both the GFP (green) and DAPI (blue) filters. All images were taken at the same intensity setting. Oocytes were separated into three groups based on the cortical granule distribution pattern. Pattern I was characterized by the presence of CGs in clusters or large aggregates. In pattern II, the CGs were individually dispersed and partially clustered, and in pattern III, the CGs were completely dispersed [33].

### 2.5. Statistical Analysis

Statistical analysis for all methods was conducted using Jamovi 1.6 (the Jamovi Project). Polar body extrusion data were analyzed using a one-way ANOVA. Immature nuclear staining, cortical granule staining, and mitochondrial distribution were analyzed using a generalized multinomial model (GMM), and all other data were analyzed using a generalized linear model (GLM) with post hoc tests. Data were considered statistically significant if *p* < 0.05.

## 3. Results

### 3.1. Nuclear Maturation

Overall, nuclear maturation as determined by polar body extrusion was significantly higher in FLI-matured oocytes compared to BMM oocytes at 75.1 ± 0.98% (2355/3131) vs. 66.8 ± 1.09% (2084/3130), respectively (40 replicates; *p* < 0.001).

#### 3.1.1. Immature Oocyte Nuclear Staining

Immature oocytes are disproportionately arrested during in vitro maturation (Table 1, Figure S1). The FLI group had a significantly lower percentage of GV oocytes at 2.16 ± 1.39% (4/133) compared to the BMM group at 12.5 ± 3.13% (21/153) (*p* < 0.01). The FLI group also had a significantly greater percentage of telophase I oocytes at 36.2 ± 3.76% (46/133) compared to the BMM group at 18.6 ± 2.73% (27/153) (*p* < 0.05).

#### 3.1.2. Meiotic Spindle Staining

**Figure 1:**
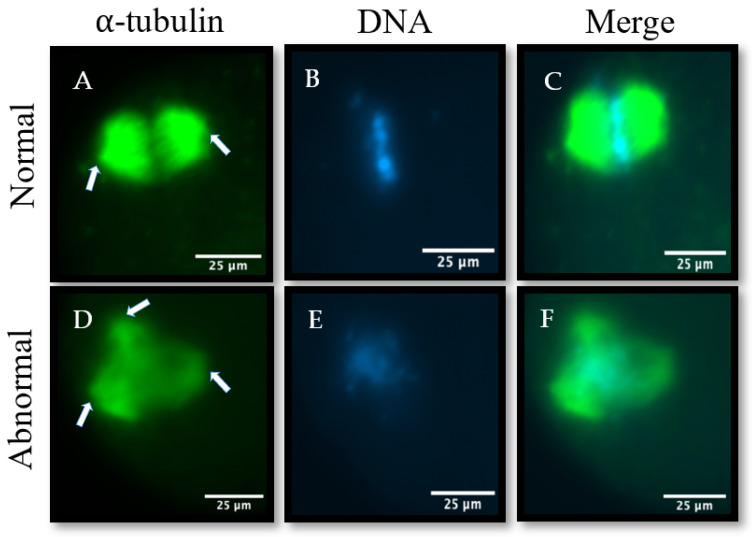
Representative images of normal (**A**–**C**) and abnormal (**D**–**F**) meiotic spindles within MII oocytes. Microtubules were stained with α-tubulin (**A**,**D**), and chromosomes were stained with Hoechst 33342 (**B**,**E**). Arrows indicate meiotic spindle poles. Two poles indicate a normal meiotic spindle (**A**), and greater than two poles indicate an abnormal spindle (**D**). Normal meiotic spindles have barrel-shaped microtubules with aligned chromosomes, and abnormal meiotic spindles have misshapen or misaligned microtubules with disorganized chromosomes. All images were taken at 200× magnification.

Representative images of normal and abnormal meiotic spindles are shown in Figure 1. The FLI group had a significantly higher percentage of normal meiotic spindles compared to the BMM group at 68.5 ± 6.51% (22/32) vs. 50.0 ± 2.27% (16/32), respectively (*p* < 0.001). Therefore, FLI supplementation in bovine maturation media improved nuclear progression through meiosis I and increased proper meiotic spindle formation.

### 3.2. Cytoplasmic Maturation

#### 3.2.1. Mitochondrial Distribution and Mass

**Figure 2:**
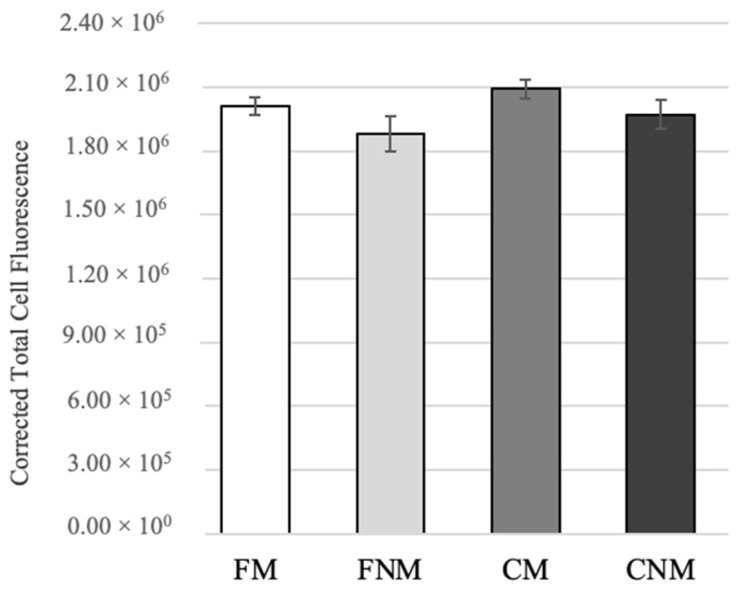
Mean mitochondrial mass after in vitro maturation as determined by MitoTracker^®^ Green fluorescent staining intensity. Five replicates of staining were performed with 70 total oocytes for FM, 29 total oocytes for FNM, 65 total oocytes for CM, and 31 total oocytes for CNM. The data were analyzed with a generalized linear model with a post hoc test.

**Figure 3:**
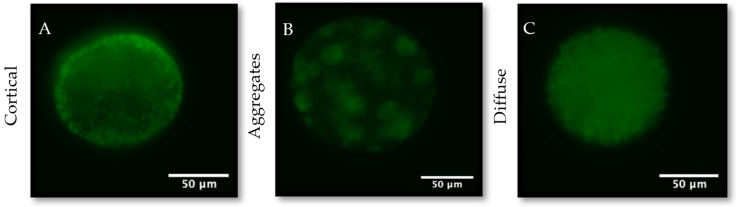
Representative images of cortical (**A**), aggregates (**B**), and diffuse (**C**) mitochondrial distribution in bovine oocytes as assessed by MitoTracker^®^ Green staining. During bovine oocyte maturation, mitochondria must rearrange from a cortical to a diffuse distribution to function properly. All images were taken at 200× magnification.

No significant differences were observed in mitochondrial mass (Figure 2, *p* > 0.05). Mitochondrial distribution was assessed by placing oocytes into groups with one of three distribution patterns: diffuse, aggregates, or cortical (Figure 3). No significant differences in mitochondrial distribution were observed between CM and CNM oocytes (Table 2). The percentage of oocytes exhibiting a diffuse distribution of mitochondria was significantly higher in FM compared to CM oocytes at 88.3 ± 3.77% vs. 76.7 ± 3.96%, respectively (*p* < 0.05). Inversely, the percentage of oocytes with cortical distribution was significantly lower in FM at 11.0 ± 3.23% compared to CM oocytes at 18.3 ± 3.81% (*p* < 0.05). FNM had a significantly lower incidence of diffuse distribution at 59.3 ± 6.34% compared to FM at 88.3 ± 3.77% (*p* < 0.001), CM at 76.7 ± 3.96% (*p* < 0.05), and CNM at 77.0 ± 4.94% (*p* < 0.05). Conversely, the incidence of cortical distribution was significantly higher in FNM at 23.3 ± 3.19% compared to FM at 11.0 ± 3.23% (*p* < 0.01), CM at 18.3 ± 3.81% (*p* < 0.05), and CNM at 19.0 ± 3.63% (*p* < 0.05). The aggregate mitochondrial distribution incidence was similar in all groups.

#### 3.2.2. Mitochondrial Membrane Potential (MMP)

**Figure 4:**
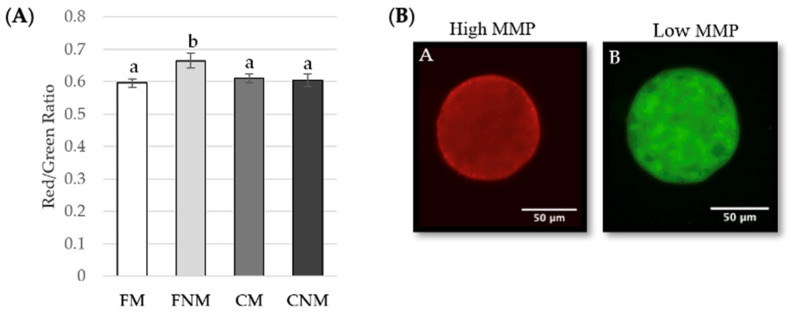
Effect of FLI medium on mitochondrial membrane potential after in vitro maturation as determined by JC-1 staining. (**A**) Mean red (high potential)/green (low potential) ratio of FM (n = 95), FNM (n = 44), CM (n = 95), and CNM (n = 45) oocytes. ^a,b^ superscripts represent significant differences (*p* < 0.05). Four replicates were analyzed with a generalized linear model with a post hoc test. (**B**) Representative images of JC-1 staining. At high potentials, mitochondria form JC-1 aggregates, which can be visualized with red fluorescence (A). At low potentials, mitochondria form JC-1 monomers, which can be visualized with green fluorescence (B). Images were taken at 200× magnification.

The activity of mitochondria as well was evaluated within the four oocyte groups. Mitochondrial activity was determined by assessing mitochondrial membrane potential (MMP) (Figure 4A). High membrane potential is associated with higher mitochondrial activity, and vice versa (Figure 4B). MMP was significantly higher in FNM oocytes compared to FM (*p* < 0.01), CM (*p* < 0.05), and CNM (*p* < 0.05).

#### 3.2.3. Mitochondrial DNA Content

**Figure 5:**
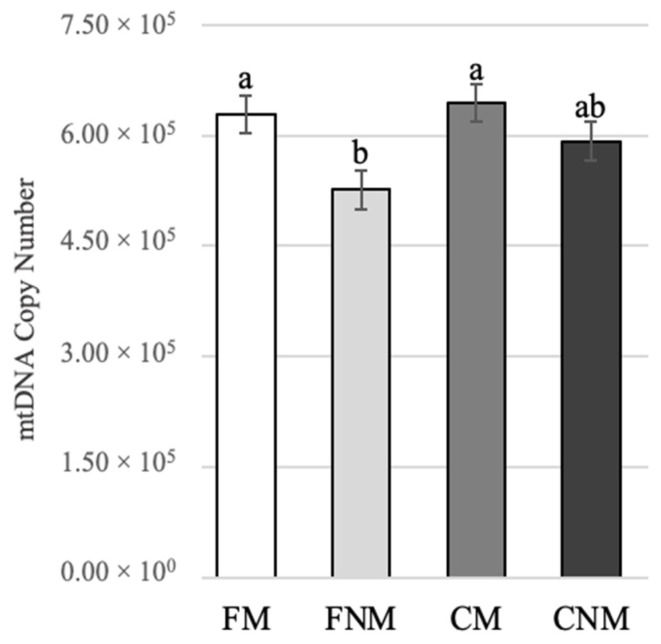
Mean mitochondrial DNA copy number after in vitro maturation as determined by RT-qPCR in FM (n = 74), FNM (n = 76), CM (n = 74), and CNM (n = 74) oocytes. ^a,b^ superscripts represent significant differences (*p* ≤ 0.01). Six replicates were analyzed with a generalized linear model with a post hoc test.

The amount of mitochondrial DNA (mtDNA) was evaluated within the four oocyte groups. mtDNA copy number followed an inverse pattern to MMP (Figure 5). The mtDNA copy number was significantly lower in FNM compared to both FM (*p* < 0.05) and CM oocytes (*p* < 0.01). Mitochondrial dynamics differ between FLI-supplemented and control oocytes.

#### 3.2.4. Cortical Granule Distribution

**Figure 6:**
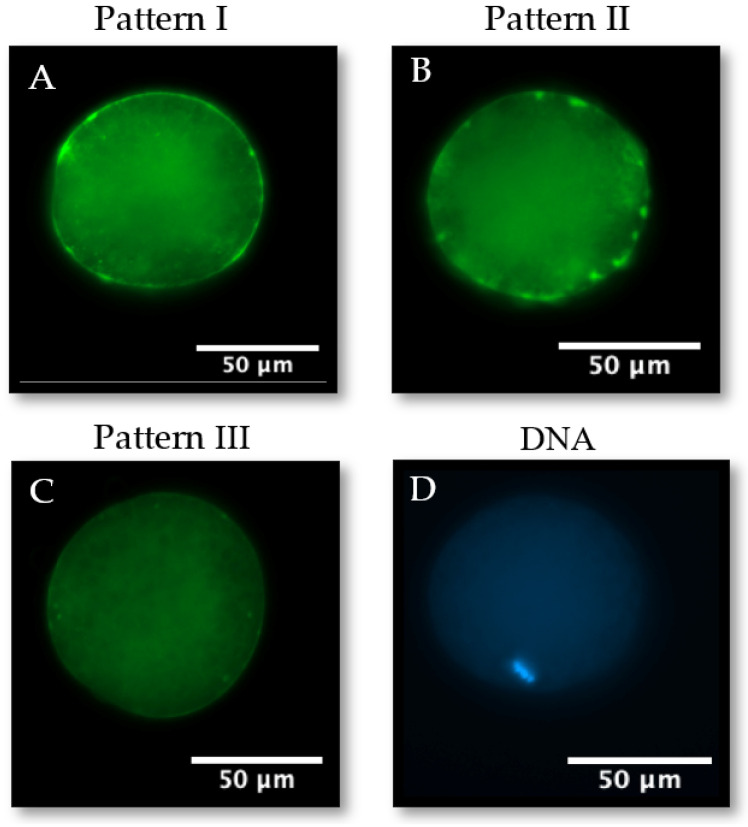
Representative images of cortical granule (GC, FITC-LCA; (**A**–**C**)) and chromosome (Hoechst 33342; (**D**)) staining in MII bovine oocytes. All images were taken at 200× magnification. Pattern I (**A**): CGs in clusters or aggregates in the cortex. Pattern II (**B**): CGs individually dispersed out of the cortex and partially clustered in the cortex. Pattern III (**C**): CGs completely dispersed out of the cortex.

In total, there were 160 oocytes from 4 replicates used in FITC-LCA staining to identify cortical granule distribution. Representative images of the three cortical granule distributions—pattern I, pattern II, or pattern III—that oocytes were assigned to are shown in Figure 6. Oocytes matured in the FLI-supplemented medium had a significantly higher incidence of pattern I at 44.3 ± 7.20% than BMM at 25.9 ± 4.63% (*p* < 0.05) (Table 3). No significant differences were observed in patterns II or III. FLI supplementation improved cortical granule redistribution during bovine oocyte IVM.

## 4. Discussion

Proper nuclear maturation and interrelated cytoplasmic maturation are essential for the production of developmentally competent oocytes. We previously reported significant improvements in oocyte maturation and both blastocyst development following IVF and SCNT and SCNT in vivo development to term using oocytes derived from FLI-supplemented maturation medium [12]. In this study, we observed differences in specific nuclear and cytoplasmic maturation events between FLI and BMM-matured oocytes.

During in vitro maturation, the bovine oocyte must undergo germinal vesicle breakdown and meiosis I, arresting at metaphase of meiosis II [34]. More FLI-matured oocytes progressed through meiosis I during IVM than BMM-matured oocytes, as indicated by the lower percentage of GV oocytes, the higher percentage of telophase I oocytes, and, most importantly, the higher incidence of FLI oocytes reaching MII stage based on polar body extrusion assessment. These data together suggest that FLI supplementation aids in vitro matured oocytes in achieving germinal vesicle breakdown, completing meiosis I, and arresting at metaphase of meiosis II. During this nuclear maturation, proper meiotic spindle assembly must take place. The spindle assembly checkpoint ensures that the genetic DNA found on chromosomes is being separated properly by microtubules during meiosis [35]. Staining of the meiotic spindle revealed that FLI oocytes have a higher occurrence of normal meiotic spindle assembly compared to BMM oocytes. Barrel-shaped microtubules and properly aligned chromosomes in the meiotic spindle are necessary for accurate chromosome separation during meiosis II to avoid any genetic deformities in the developing embryos after fertilization [36]. If the meiotic spindle is assembled improperly within the oocyte, subsequent embryos may undergo abnormal growth, leading to arrest at the early stages of in vitro development. This result could explain our previously observed improvement in blastocyst development following in vitro fertilization using oocytes matured in FLI-supplemented medium [12].

Meiotic spindle formation during IVM is a process that requires ample mitochondrial activity [37]. The non-genetic component of the meiotic spindle is the microtubules, which help align and separate the chromosomes, as stated above. However, microtubules are dynamic and have been implicated in the redistribution of organelles, such as mitochondria, during in vitro maturation [38]. Proper rearrangement of both mitochondria and cortical granules is needed within the oocyte during IVM. Out of all mitochondrial components analyzed in this study, mitochondrial redistribution was the only one that showed a significant difference between FLI or BMM-matured oocytes. Mitochondrial distribution changing from cortical, which presents itself during the GV stage, to diffuse throughout the cytoplasm in metaphase II oocytes during bovine in vitro maturation has been associated with better embryo development [27]. There was a significantly higher incidence of diffuse and a lower incidence of cortical mitochondrial distribution observed between FLI MII (FM) oocytes and control MII (CM) oocytes after in vitro maturation. FLI supplementation enhanced this necessary mitochondrial redistribution, which likely contributed, in part, to the increased embryo development after both IVF and SCNT [12].

Besides mitochondrial distribution, we also observed decreased mitochondrial activity in both FM and CM oocytes compared to FNM oocytes. High mitochondrial activity may be needed during certain stages of bovine oocyte in vitro maturation to obtain both proper cytoplasmic and nuclear maturation. It has been found that an increase in mitochondrial activity happens twice in bovine oocytes with greater developmental competence during IVM [21]. One of the increases in mitochondrial activity happens immediately before the completion of maturation, which may explain our results of higher mitochondrial activity in FNM oocytes, as a high percentage of these were in the telophase I phase of meiosis. The high mitochondrial activity seen in FNM oocytes might also be the reason behind increased meiotic spindle formation, increased polar body extrusion, and proper mitochondrial redistribution in FM oocytes. Another explanation for the increase in FNM oocytes’ MMP may be that mitochondrial fusion during IVM is increased by FLI supplementation. Specifically, IGF1 has been shown to activate AMP-activated protein kinase (AMPK), and upregulation of AMPK increases mitochondrial fusion [39,40]. Mitochondrial fusion is necessary for proper mitochondrial function under stress conditions, which are present during IVM of oocytes [41]. An imperative prerequisite for mitochondrial fusion is an increase in mitochondrial membrane potential [42]. Thus, increased MMP in FNM oocytes may be due to FLI supplementation mitigating environmental stress throughout IVM to produce higher-quality MII oocytes.

Our finding of the decreased mtDNA content in FNM oocytes compared to FM and CM oocytes aligns with the findings of Mao et al., where MII stage oocytes had significantly higher mtDNA copy numbers compared to immature oocytes [43]. MtDNA is highly susceptible to damage caused by reactive oxygen species (ROS) because it is in close contact with the electron transport chain, where most ROS are produced [44]. If an oocyte has too many mitochondria or the mitochondria present are dysfunctional, then there is an increased risk of reactive oxygen species buildup, which could lead to DNA strand breaks [45]. Since FNM oocytes have higher mitochondrial activity, they may have higher ROS and, thus, lower mtDNA than FM and CM oocytes. However, mitochondrial mass was similar in all groups. Most likely, FLI supplementation does not cause an increase or decrease in mitochondrial mass because either scenario could be detrimental to the oocyte and subsequent embryo development. An increase could potentially cause an increase in ROS within the oocyte due to a higher number of mitochondria undergoing oxidative phosphorylation, which produces about 90% of cellular ROS [46]. Similarly, a decrease in the mitochondrial population may create a roadblock to oocyte maturation and embryo development, as ATP production is essential for both [18].

Cortical granule (CG) redistribution during IVM is also an important element of bovine cytoplasmic maturation in oocytes. CGs must go from diffuse to large clusters or aggregates around the oocyte cortex to block polyspermy during fertilization via exocytosis [47]. There was an increase in pattern I, which is characterized by large clusters of CGs around the cortex, in the FLI-matured oocytes compared to the BMM-matured oocytes. These data present another clue into why FLI-matured oocytes that undergo IVF develop better than BMM-matured ones. Because there are no clear differences between CM and CNM oocytes for any of the mitochondrial assessments performed, some CNM oocytes may undergo the cytoplasmic reorganization needed during IVM but are prevented from achieving nuclear maturation at the same time. Thus, FLI supplementation seems to help coordinate both nuclear and cytoplasmic maturation during in vitro bovine oocyte maturation.

The mechanistic pathways that FLI works through to increase both nuclear and cytoplasmic maturation in bovine oocytes are most likely those that have been elucidated with the addition of each cytokine individually. However, with the addition of all three cytokines together, these pathways are presumably upregulated to a greater degree, especially because they are all essential for oocyte maturation and are interconnected. FGF2 supplementation has been implicated in the regulation of cumulus cell expansion through activation of the c-Mos/mitogen-activated protein kinase (MAPK) pathway and oocyte meiosis through an increase in maturation-promoting factor (MPF) [7]. Unlike FGF2, LIF acts through two signaling cascades, MAPK3/1 and the signal transducer and activator of transcription 3 (STAT3)-dependent pathways, but is more heavily controlled by JAK/STAT3 [8]. Similarly, IGF1 acts on both the oocyte as well as the embryo, increasing oocyte quality and embryonic development through the phosphoinositide 3-kinase (PI3K)/protein kinase B (AKT)/mammalian target of rapamycin (mTOR) pathway [9,10].

The c-Mos/MAPK pathway within the oocyte is needed for the oocyte to undergo meiosis I and arrest at metaphase of meiosis II through MPF activation [48]. An increase in this pathway could be the reason behind increased polar body extrusion and proper meiotic spindle assembly. An increase in the c-Mos/MAPK pathway through the synergistic actions of both FGF2 and LIF is most likely the reasoning behind increased meiosis I advancement and nuclear maturation in FLI-supplemented bovine oocytes. The JAK/STAT pathway is started by a cytokine-receptor interaction on the cell surface and works to enhance gene transcription within the nucleus. It has been shown to increase numerous genes, including Bcl-2, c-Myc, AOX, and GFAP, which are involved in anti-apoptosis, cell-cycle progression, lipid metabolism, and differentiation, respectively [49]. Any mitochondrial activity or distribution changes in FLI-supplemented bovine oocytes most likely impact these cellular processes, as mitochondria play a key role in all four of them. The PI3K/AKT/mTOR pathway that is upregulated by IGF1 acts to increase mitochondrial activity within the oocyte as well as decrease DNA damage [50]. No differences were seen in mitochondrial activity between FM and CM oocytes, which could be due to mitochondrial activity being affected by FLI supplementation before polar body extrusion, as can be seen by the increase in FNM but not CNM oocytes. AKT and mTOR are two essential proteins in charge of meiotic spindle assembly, as they both aid in microtubule assembly and function. Furthermore, AKT is essential for early embryo development [51]. An increase in AKT may be part of the reasoning behind increased embryo development after both IVF and SCNT using FLI-supplemented oocytes. However, this has yet to be explored. The PI3K/AKT/mTOR pathway, coupled with the c-Mos/MAPK pathway, is most likely responsible for the increase in proper meiotic spindle assembly in FLI-supplemented bovine oocytes. Because this pathway affects microtubules, which are necessary for organelle and vesicle movement within the oocyte, we also believe that it may be responsible for the proper redistribution of both mitochondria and cortical granules within FLI-supplemented bovine oocytes. Many specifics of FLI-supplementation mechanisms are yet to be elucidated; however, this research proves there are both nuclear and cytoplasmic differences within FLI-supplemented bovine oocytes that are most likely linked to the aforementioned pathways, which are increased during individual supplementation of these cytokines.

## 5. Conclusions

In conclusion, FLI supplementation in bovine oocyte maturation medium improved nuclear progression through meiosis I and increased proper meiotic spindle formation, as well as both mitochondrial and cortical granule redistribution. These findings indicate that FLI supplementation during bovine IVM improved the coordination of nuclear and cytoplasmic maturation in the oocytes. Future studies should focus on elucidating the specific mechanisms through which FLI works to achieve proper nuclear and cytoplasmic coordination, which may be part of the signaling pathways triggered during supplementation of each cytokine, leading to an increase in the efficiency of assisted reproductive techniques in livestock and potentially humans.

## Figures and Tables

**Table 1:** Effect of FLI supplementation on meiosis progression during IVM.

Group	Nuclear Stage				
	GV	GVBD	Metaphase I	Anaphase I	Telophase I
**FLI**	4/133 (2.16 ± 1.39%) ^a^	29/133 (21.5 ± 2.43%) ^a^	31/133 (23.0 ± 2.13%) ^a^	23/133 (17.1 ± 2.48%) ^a^	46/133 (36.2 ± 3.76%) ^a^
**BMM**	21/153 (12.5 ± 3.13%) ^b^	36/153 (24.2 ± 2.20%) ^a^	36/153 (23.2 ± 2.97%) ^a^	33/153 (21.6 ± 1.29%) ^a^	27/153 (18.6 ± 2.73%) ^b^

^a,b^ superscripts indicate significant differences. (*p* < 0.01 GV; *p* < 0.05 Telophase I); 7 replicates; mean ± SEM; generalized multinomial model with post hoc test.

**Table 2:** Effect of FLI supplementation on mitochondrial distribution.

Group	Mitochondrial Distribution		
	Diffuse	Aggregates	Cortical
**FM**	121/138 (88.3 ± 3.77%) ^a^	1/138 (0.69 ± 0.69%) ^a^	16/138 (11.0 ± 3.23%) ^a^
**FNM**	37/60 (59.3 ± 6.34%) ^b^	10/60 (17.4 ± 6.27%) ^a^	13/60 (23.3 ± 3.19%) ^b^
**CM**	107/138 (76.7 ± 3.96%) ^c^	7/138 (5.04 ± 1.33%) ^a^	24/138 (18.3 ± 3.81%) ^c^
**CNM**	49/64 (77.0 ± 4.94%) ^ac^	3/64 (4.03 ± 2.88%) ^a^	12/64 (19.0 ± 3.63%) ^ac^

^a,b,c^ superscripts indicate significant differences. (*p* < 0.05); 8 replicates; mean ± SEM, generalized multinomial model with a post hoc test.

**Table 3:** Effect of FLI supplementation on cortical granule distribution.

Group	Cortical Granule Distribution		
	Pattern I	Pattern II	Pattern III
**FLI**	34/76 (44.3 ± 7.20%) ^a^	34/76 (42.1 ± 8.51%) ^a^	8/76 (13.6 ± 5.70%) ^a^
**BMM**	23/84 (25.9 ± 4.63%) ^b^	46/84 (54.6 ± 3.37%) ^a^	15/84 (19.5 ± 4.79%) ^a^

^a,b^ superscripts indicate significant differences. (*p* < 0.05); 4 replicates; mean ± SEM; generalized linear model with a post hoc test.

## Data Availability

All data generated and analyzed during this study are available from the corresponding author upon reasonable request.

## Supplementary Materials

The following supporting information can be downloaded at: https://www.mdpi.com/article/10.3390/ani14121837/s1, Figure S1: Representative images of immature oocyte nuclear staining. Nuclear maturation during IVM progresses as follows: GV (A), GVBD (B), MI (C), Anaphase I (D), Telophase I (E), ending in metaphase II and polar body extrusion (mature oocytes).
