# Supplementary material for: Cytokine-Supplemented Maturation Medium Enhances Cytoplasmic and Nuclear Maturation in Bovine Oocytes
Source: Animals (Basel). 2024 Jun 20;14(12):1837. doi: 10.3390/ani14121837 (PMC11200980; doi:10.3390/ani14121837)
Supplement: Supplementary file 1 [file animals-14-01837-s001.zip › animals-3036797-supplementary.pdf]

animals

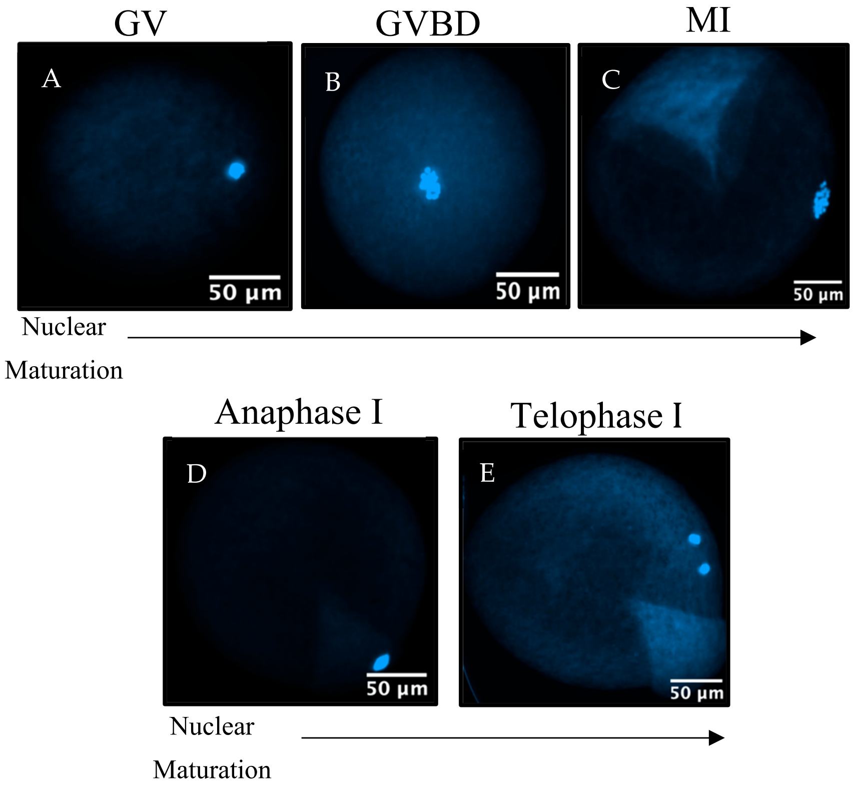

Supplementary Figure S1. Representative images of immature oocyte nuclear staining. Nuclear maturation during IVM progresses as follows: GV (A), GVBD (B), MI (C), Anaphase I (D), Telophase I (E), ending in metaphase II and polar body extrusion (mature oocytes).
